# Effect of Cause-of-Death Training on Agreement Between Hospital Discharge Diagnoses and Cause of Death Reported, Inpatient Hospital Deaths, New York City, 2008–2010

**DOI:** 10.5888/pcd12.140299

**Published:** 2015-01-15

**Authors:** Paulina Ong, Melissa Gambatese, Elizabeth Begier, Regina Zimmerman, Antonio Soto, Ann Madsen

**Affiliations:** Author Affiliations: Paulina Ong, Melissa Gambatese, Elizabeth Begier, Regina Zimmerman, Antonio Soto, Ann Madsen, New York City Department of Health and Mental Hygiene, New York, New York.

## Abstract

**Introduction:**

Accurate cause-of-death reporting is required for mortality data to validly inform public health programming and evaluation. Research demonstrates overreporting of heart disease on New York City death certificates. We describe changes in reported causes of death following a New York City health department training conducted in 2009 to improve accuracy of cause-of-death reporting at 8 hospitals. The objective of our study was to assess the degree to which death certificates citing heart disease as cause of death agreed with hospital discharge data and the degree to which training improved accuracy of reporting.

**Methods:**

We analyzed 74,373 death certificates for 2008 through 2010 that were linked with hospital discharge records for New York City inpatient deaths and calculated the proportion of discordant deaths, that is, death certificates reporting an underlying cause of heart disease with no corresponding discharge record diagnosis. We also summarized top principal diagnoses among discordant reports and calculated the proportion of inpatient deaths reporting sepsis, a condition underreported in New York City, to assess whether documentation practices changed in response to clarifications made during the intervention.

**Results:**

Citywide discordance between death certificates and discharge data decreased from 14.9% in 2008 to 9.6% in 2010 (*P* < .001), driven by a decrease in discordance at intervention hospitals (20.2% in 2008 to 8.9% in 2010; *P* < .001). At intervention hospitals, reporting of sepsis increased from 3.7% of inpatient deaths in 2008 to 20.6% in 2010 (*P* < .001).

**Conclusion:**

Overreporting of heart disease as cause of death declined at intervention hospitals, driving a citywide decline, and sepsis reporting practices changed in accordance with health department training. Researchers should consider the effect of overreporting and data-quality changes when analyzing New York City heart disease mortality trends. Other vital records jurisdictions should employ similar interventions to improve cause-of-death reporting and use linked discharge data to monitor data quality.

## Introduction

Mortality statistics, derived from death certificates, summarize population disease burden, inform research and program evaluations, and help determine public health priorities (1,2). Inaccurate cause-of-death reporting may result in misinformed policies, programs, and research (3–5). Previous studies found low levels of agreement between the death certificate cause of death and the sequence of events reported on the medical record (2,6,7). One possible reason for this disagreement is inadequate training of health care providers in death certification (8,9). Clinicians throughout the United States overreport heart disease as a cause of death and do so to a greater degree in New York City. A 2001 study of 4 US cities found that medical records did not substantiate 20% of death certificates that reported heart disease as an underlying cause of death (10), whereas a similar 2003 New York City study found an average discrepancy of 33% (11).

Training can improve the accuracy of cause-of-death reporting (12,13). Since 2008, the New York City Department of Health and Mental Hygiene (DOHMH) has produced numerous cause-of-death training resources, including written materials (14), Web-based modules (15), and a hospital intervention with staff in-service trainings (16,17). The training intervention was launched in 2009 at 8 noncardiac-specialty hospitals, each of which had reported more than 60% of deaths as due to heart disease. The main training objective was to teach and review methodology for accurate cause-of-death reporting on the death certificate. Course materials also emphasized legal requirements for reporting and the importance of valid documentation. Heart disease deaths at these hospitals fell from 68.8% in 2008 to 32.4% in 2010; an increase in other causes was generally proportional to their burden in the population (16,18). Clinicians reported at DOHMH trainings that the New York City medical examiner’s policy of not allowing sepsis to stand alone as an underlying cause of death (14) had been misinterpreted by hospital staff to mean that the terms “sepsis” or “septicemia” were not accepted for use anywhere on the death certificate.

The objective of our study was to assess the degree to which death certificates citing heart disease as cause of death agreed with hospital discharge data, which were used in validation studies as a proxy for the medical record (3,19); the discharge diagnosis associated with lack of agreement; and whether New York City’s cause-of-death training efforts improved agreement. More generally, we evaluated the feasibility of using linked hospital discharge data and death certificate records for future initiatives to improve data quality.

## Methods

### Intervention hospitals

Our analysis included the 8 intervention hospitals targeted in the 2009 New York City DOHMH training intervention. These hospitals had among the highest ratios of reported heart disease deaths to total deaths (16). Nonintervention hospitals included the 50 remaining hospitals reporting inpatient deaths in 2009. Additional characteristics of intervention and nonintervention hospitals have been described previously (11,16,18). For our analysis, we defined “preintervention” as the period from January 1 through December 31, 2008, and “postintervention” as the period from January 1 through December 31, 2010.

### Data sources and key variables

**Death certificates and cause of death.** The New York City Health Code mandates complete and accurate reporting of all deaths that occur in the city (20). The death certificate contains detailed information about the decedent as well as the circumstances and causes of death. Information about immediate, intermediate, underlying, and contributing causes of death are solicited from clinicians in conformance with the 2003 US Standard Certificate of Death (21).

The National Center for Health Statistics’ (NCHS’s) Mortality Medical Data System software assigns the corresponding International Classification of Disease, 10th Revision (ICD-10) code (22) to each condition, event, or injury reported as contributing to death. The software then applies a standardized algorithm to assign an underlying cause to each death. A trained New York City DOHMH nosologist assigns causes manually when automatic coding fails (21).

The World Health Organization defines the underlying cause of death as the “disease or injury which initiated the train of morbid events leading directly to death, or the circumstances of the accident which produced the fatal injury” (23). We flagged deaths as due to heart disease if the ICD-10 code for the underlying cause of death was I00–I09, I11, I13, or I20–I51, in accordance with NCHS definitions (24).

**Hospital discharge records and diagnosis codes.** The New York Statewide Planning and Research Cooperative System (SPARCS) maintains a database of patient-level diagnoses and treatments of every hospital discharge, as mandated by public health law (25). Medical coders review the medical record to determine a principal diagnosis, defined as the “condition established after study to have been chiefly responsible for occasioning the admission of the patient to the hospital for care,” as well as other diagnoses, which include “all conditions that coexisted at admission, or developed subsequently, which affected the treatment received or length of stay” (26).

SPARCS diagnoses are coded according to the International Classification of Disease, 9th Revision, Clinical Modification (ICD-9-CM) (27). To compare SPARCS diagnoses and underlying causes of death, we created a cross reference between ICD-9-CM and ICD-10 codes for heart disease and other conditions (Box).

**Box. International Classification of Disease Codes, 9th and 10th Editions, With Disease Classes**

**Table:** 

ICD 10 Codes	ICD 9–CM Codes	Disease Class
A00-B99	001–139	Infectious and parasitic diseases
C00-D48	140–239	Neoplasms
D50-E90	240–289	Endocrine, nutritional, and metabolic diseases, and immunity disorders; diseases of the blood and blood-forming organs
F00-F99	290–319	Mental disorders
G00-G99	320–389	Diseases of the nervous system and sense organs
I00-I99	390–459	Diseases of the circulatory system
J00-J99	460–519	Diseases of the respiratory system
K00-K93	520–579	Diseases of the digestive system
N00-N99	580–629	Diseases of the genitourinary system
O00-O99	630–679	Complications of pregnancy, childbirth, and puerperium
L00-L99	680–709	Diseases of the skin and subcutaneous tissue
M00-M99	710–739	Diseases of the musculoskeletal system and connective tissue
Q00-Q99	740–759	Congenital anomalies
P00-P96	760-779	Certain conditions originating in the perinatal period
R00-R99	780-799	Symptoms, signs, and ill-defined conditions
S00-T98	800-999	Injury and poisoning

**Linked data set.** The New York State Bureau of Biometrics and Health Statistics matched SPARCS records to New York City death records by using a combination of deterministic and probabilistic methods. Almost all (98.5%) SPARCS records with a disposition of death were matched in 2004, and in 2005, 90.7% of matches met criteria specified for a near-zero probability of a false match (28).

We analyzed linked data for New York City inpatient, natural-cause deaths that occurred from 2008 through 2010 by comparing diagnoses from the hospital discharge record with the underlying cause on the death certificate to identify unsubstantiated reports of heart disease. For this study, we focused on inpatient deaths, because our intervention took place only at hospitals. We also limited our analysis to hospital admissions during which the patient died. Our final sample included 74,373 inpatient deaths, approximately 25,000 for each year of study, and reflected 46.6% of all deaths that occurred in New York City from 2008 through 2010. Our sample represented 91.3% of inpatient deaths. Unmatched deaths probably resulted from misaligned identifiers or misreported place-of-death information. Nonetheless, our matched sample closely represented reporting of heart disease for New York City inpatient deaths, because 29.3% of all inpatient death certificates and 28.8% of matched inpatient death certificates reported an underlying cause of heart disease (Table).

**Table:** Inpatient Deaths Reported on the Death Certificate and Disagreement with the Hospital Discharge Record, Heart Disease Deaths, New York City 2008–2010

Deaths Reported	2008	2009	2010
**New York City deaths, all causes, n**	54,193	52,881	52,575
Death certificates reporting heart disease as underlying cause, n (%)	21,192 (39.1)	20,086 (38.0)	17,929 (34.1)
**New York City inpatient deaths, n**	28,408	26,949	26,097
Death certificates reporting heart disease as underlying cause, n (%)	8,334 (29.3)	7,573 (28.1)	5,607 (21.5)
Intervention hospitals, n	2,857	2,504	1,254
Nonintervention hospitals, n	5,477	5,069	4,353
**New York City inpatient deaths successfully matched to a hospital discharge record, n**	25,637	24,601	24,135
Death certificates reporting heart disease as underlying cause, n (%)	7,373 (28.8)	6,846 (27.8)	5,163 (21.4)
Intervention hospitals, n	2,703	2,361	1,199
Nonintervention hospitals, n	4,670	4,485	3,964
**New York City inpatient deaths matched to a hospital discharge record with no heart disease diagnosis on discharge record, n (%)^a^**	1,096 (14.9)	934 (13.6)	496 (9.6)^d^
Intervention hospitals, n (%)^b^	547 (20.2)	438 (18.6)	107 (8.9)^d^
Nonintervention hospitals, n (%)^c^	549 (11.8)	496 (11.1)	389 (9.8)^d^

a Calculated as a percentage of matched New York City inpatient death certificates reporting heart disease as an underlying cause of death

b Calculated as a percentage of matched New York City inpatient death certificates reporting heart disease as the underlying cause at intervention hospitals

c Calculated as a percentage of matched New York City inpatient death certificates reporting heart disease as the underlying cause at nonintervention hospitals

d The change in proportion of matched inpatient records with no mention of heart disease on the discharge record between 2008 and 2010 was statistically significant at *P* = .01. *P* values were calculated using a 2-tailed *z* test for proportions.

### Analysis

The focus of our study was agreement between the underlying cause of death reported on the death certificate and the principal and other diagnoses listed on the hospital discharge record. We defined a discordant record as one in which the death certificate reported an ICD-10 underlying cause of death of heart disease and the SPARCS record had no corresponding ICD-9-CM diagnosis for heart disease. We calculated the proportion of discordant records by year and intervention hospital status and tested statistical significance for changes from 2008 through 2010 using a *z* test for proportions. Cited probabilities are 2-tailed, and a value of *P* < .01 was considered significant because of the large size of the population.

Among discordant reports, we conducted a secondary analysis of the principal diagnosis to identify conditions that were important to death but misrepresented by a reported underlying cause of heart disease. We summarized top principal diagnoses among discordant reports by year, grouped into ICD-9-CM disease classes (Box).

We calculated post hoc the proportion of all inpatient death certificates that reported sepsis as an immediate, intermediate, or underlying cause of death to assess whether reporting practices changed in response to clarifications made during the intervention. We defined sepsis by the NCHS classification of septicemia (ICD-10 codes A40, A41) (24). All analyses were conducted using SAS version 9.2 (SAS Institute, Inc).

## Results

### Heart disease mortality and discordant reports of heart disease deaths

**Figure 1:**
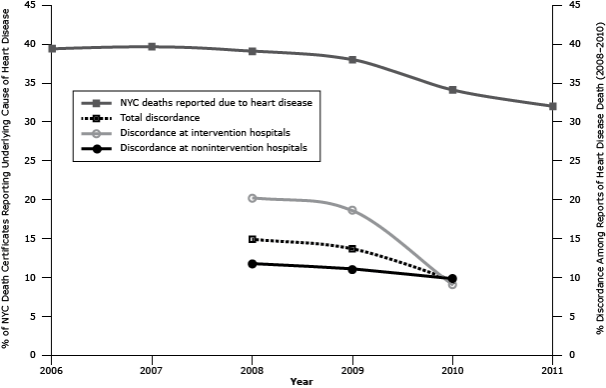
Five-year trend in death certificates reporting heart disease as an underlying cause of death (2006–2011) and 3-year trend in discordance among reports of heart disease deaths^a^ (2008–2010) in New York City. Abbreviation: NA, not applicable. We defined discordant reports of heart disease as deaths for which the death certificate reported an underlying cause of death of heart disease and the Statewide Planning and Research Cooperative System hospital discharge record had no corresponding diagnosis of heart disease. **Table:** 

Heart disease was the reported underlying cause of death on 39.1% (n = 21,192) of all New York City death certificates in 2008, 38.0% (n = 20,086) in 2009, and 34.1% (n = 17,929) in 2010 (Table). The proportion of inpatient death certificates reporting heart disease declined from 29.3% (n = 8,334) in 2008 to 28.1% (n = 7,573) in 2009 and 21.5% (n = 5,607) in 2010. Proportions were similar among deaths matched to a SPARCS record: 28.8% (n = 7,373) in 2008, 27.8% (n = 6,846) in 2009, and 21.4% (n = 5,163) in 2010. In 2008, 14.9% (n = 1,096) of the 7,373 citywide matched inpatient heart disease deaths were discordant. Total discordance fell to 13.6% (n = 934) in 2009 and 9.6% (n = 496) in 2010 (Figure 1). Among intervention hospitals, discordance fell from 20.2% (n = 547) in 2008 to 18.6% (n = 438) in 2009 and 8.9% (n = 107) in 2010. Among nonintervention hospitals, discordance was 11.8% (n = 549) in 2008, 11.1% (n = 496) in 2009, and 9.8% (n = 389) in 2010. This translates to a 5.3% increase in agreement citywide, an 11.3% increase in agreement at intervention hospitals, and a 2.0% increase in agreement at nonintervention hospitals. The change in discordance from 2008 through 2010 was significant citywide (*P* < .001), at intervention hospitals (*P* < .001), and at nonintervention hospitals (*P* = .004). To facilitate comparison of trends, Figure 1 superimposes proportions of death certificates reporting an underlying cause of death of heart disease for 2006 through 2011 with proportions of discordance for 2008 through 2010.

Percentage of Discordance	2006	2007	2008	2009	2010	2011
Intervention hospitals	NA	NA	20.2	18.6	8.9	NA
Nonintervention hospitals	NA	NA	11.8	11.1	9.8	NA
Total	NA	NA	14.9	13.6	9.6	NA
Deaths reported as due to heart disease	39.4	39.7	39.1	38.0	34.1	32.0

Abbreviation: NA, not applicable.

### Principal diagnoses among discordant reports

The top 5 disease classes reported on death certificates were infectious and parasitic diseases (42.3%; n = 464); respiratory diseases (16.0%; n = 175); circulatory diseases (9.6%; n = 105); digestive diseases (7.3%; n = 80); and neoplasms (6.0%; n = 66). The overrepresentation of infectious and parasitic diseases among discordant cases prompted post hoc analyses of this class. The top principal diagnosis among discordant reports was sepsis, a condition classified within infectious and parasitic diseases, which was reported in 38.5% (n = 422) of discordant cases (90.9% of all the parasitic disease discordant cases) in 2008.

**Figure 2:**
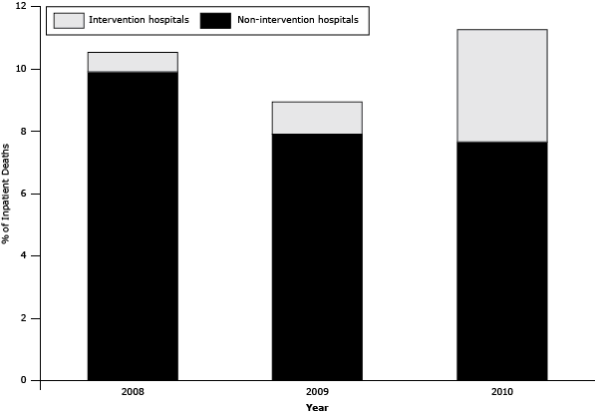
Comparison of reports of sepsis as an immediate, intermediate, or underlying cause of death on the death certificate for inpatient deaths, by intervention hospitals versus nonintervention hospitals, New York City 2008–2010. Intervention hospitals include the 8 hospitals that participated in the New York City Department of Health and Mental Hygiene’s 2009 cause-of-death reporting training intervention. **Table:** 

Because sepsis necessarily proceeds from another disease or condition and is thus not considered an appropriate underlying cause of death, post-hoc analyses included mentions of sepsis as immediate, intermediate, or underlying cause of death. The proportion of inpatient death certificates reporting sepsis increased from 2008 to 2010 (Figure 2). For inpatient deaths, sepsis reporting decreased from 10.5% of certificates (n = 2,699) in 2008 to 8.9% (n = 2,200) in 2009 and increased to 11.3% (n = 2,717) in 2010, a 0.8 percentage point increase between 2008 and 2010 (*P* = .009). At intervention hospitals, sepsis reporting increased from 3.7% (n = 157) of certificates in 2008 to 6.0% (n = 253) in 2009 and 20.6% (n = 867) in 2010, a 16.9 percentage point increase between 2008 and 2010 (*P* < .001). At nonintervention hospitals, sepsis reporting decreased from 11.9% (n = 2,542) in 2008 to 9.6% (n = 1,947) in 2009 and to 9.3% (n = 1,850) in 2010, a 2.6 percentage point decrease from 2008 through 2010 (*P* < .001) (data not shown). 

Reports of Sepsis	2008	2009	2010
Total	10.53	8.94	11.26
Intervention hospitals	0.61	1.03	3.59
Nonintervention hospitals	9.92	7.91	7.7

## Discussion

To our knowledge, ours is the first study to use hospital discharge data linked to death certificates to demonstrate the effect of training on cause-of-death reporting practices. Agreement between reporting of heart disease as an underlying cause of death and a diagnosis on the corresponding SPARCS record increased after a 2009 cause-of-death training intervention at New York City hospitals. The improvement was substantial at intervention hospitals; agreement increased 11.3%, from 79.8% in 2008 to 91.1% in 2010. Citywide, agreement increased 5.3 percentage points from 85.1% in 2008 to 90.4% in 2010. If we assume hospital discharge records are a reasonable proxy for the medical record, these results suggest the training improved the accuracy of cause-of-death reporting, which we could not conclude from the observed decline in heart disease deaths alone. The 2.0 percentage point change in agreement at nonintervention hospitals from 88.2% in 2008 to 90.2% in 2010 was also significant, potentially explained by simultaneous implementation of other lower-intensity citywide DOHMH cause-of-death training efforts (15,16).

Further evidence of the impact of the intervention on accurate cause-of-death reporting was the increase in reporting of sepsis on death certificates that paralleled the increase in agreement for heart disease deaths (Figure 2). In 2008, sepsis was the most common principal diagnosis listed for discordant cases, likely reflecting the confusion surrounding protocol for reporting sepsis. Despite clarifications made in the DOHMH City Health Information bulletin on cause-of-death reporting (14) and e-Learning course (15), rejection of stand-alone reports of sepsis appears to have resulted in misinterpretation that sepsis was not allowed to be listed as part of the cause-of-death mechanism at all. This misconception probably arose from the New York City medical examiners’ practice of not approving death certificates reporting sepsis as a stand-alone cause of death during their cremation clearance process (ie, review to approve human remains for cremation) out of concern that sepsis can at times result from an injury (14).

In cases of sepsis, hospitals apparently tended to report heart disease instead. In a survey conducted among New York City resident physicians in 2010, 70.0% of respondents believed they were forced to select an alternate cause of death when a patient died of septic shock, and among those who knowingly reported an alternate cause of death, 64.6% reported cardiovascular disease as the most frequent diagnosis assigned (9). Hospitals face pressure to expedite death registration. Inadequate training in avoiding delays through proper documentation may have resulted in staff reporting a common and uncomplicated cause of death to streamline the process. The New York City DOHMH training included detailed information on sepsis reporting and apparently enhanced understanding of reporting protocol; at intervention hospitals, sepsis was listed as a cause of death for 20.6% of deaths in 2010 compared with 3.7% in 2008. Still, sepsis remains underreported citywide. The public health significance of this data-quality issue was recently illustrated when the NYS Department of Health was unable to use death certificate data for their initiative to prevent in-hospital, sepsis-related mortality (29). Any mention of sepsis on New York City death certificates is much lower than the rest of the state, and although the gap in sepsis mortality rates between the 2 regions narrowed in 2009 and 2010, the data are not comparable for measuring the burden of sepsis mortality in New York City versus New York State hospitals (New York State Department of Health, oral communication, January 29, 2014).

These results highlight the need for cause-of-death training to improve the accuracy and validity of mortality statistics (16). The observed increased agreement between heart disease deaths and hospital discharge records corresponds to the decrease in heart disease deaths reported following the 2009 training and suggests the accuracy of heart disease reported as a cause of death increased as a result. Researchers using New York City mortality data must consider that some portion of observed declines in citywide heart disease mortality across this period reflects inflated estimates before 2008 and that observed declines are not solely due to changes in behaviors or risk factors or improved treatment. Other vital records jurisdictions can evaluate the quality of cause-of-death reporting at hospitals by using metrics such as discordance between the death certificate and hospital discharge record. These methods can serve as a reasonable proxy for resource-intensive medical record audits (19). Specifically, jurisdictions could target interventions at hospitals with poor agreement for causes of death of public health interest and monitor trends in agreement to evaluate the influence of training efforts.

A limitation of this study is that we evaluated agreement between the underlying cause of death and hospital discharge diagnoses as opposed to directly assessing accuracy of the recorded underlying cause of death through medical chart review. For our analysis, we assumed hospital discharge diagnoses were both accurate and related to death. SPARCS data are used for billing purposes so not all listed diagnoses are expected to be related to death. A single hospitalization may last weeks, and some diagnoses may relate to conditions that were stable or had resolved before death. Nonetheless, the condition reported as the cause of death would likely have been diagnosed or treated during the final admission and therefore coded in the hospital discharge record. Furthermore, it is unlikely that the extent to which SPARCS data were related to death changed during the study period, so our overall conclusions, which were based on the decreasing discordance rates over time, do not depend on this assumption.

Our definition of agreement required that the discharge record indicate a diagnosis of heart disease for deaths reporting an underlying cause of heart disease, not that any diagnosis of heart disease had to have a corresponding underlying cause of death. Thus, our analysis is conservative. These assumptions may have led to erroneous classification of cases as in agreement, resulting in an overestimation of agreement between the 2 sources. These limitations may reflect the difficulty in determining a single cause of death or selection of diagnosis codes in patients with complex medical histories. Regardless, we expect that a condition identified as the underlying cause of death should in most cases be mentioned as a diagnosis affecting a patient’s care and hospital stay.

We limited our analyses to hospital inpatient deaths because our intervention was carried out in hospitals. Therefore, our findings are not generalizable to outpatient deaths. Because not all heart disease death certificates were successfully matched, our ability to draw conclusions about all New York City inpatient heart disease deaths may be limited. However, we found that the matched data had a near-identical cause-of-death distribution to all New York City inpatient deaths, so it is unlikely that our measures of agreement were skewed by these missing observations.

We plan continued application of the data set to monitor trends in agreement between the 2 sources, extending the time period to evaluate later cause-of-death training activities and to ensure that improvement in agreement for heart disease did not occur as a consequence of practices of inaccurate reporting shifting to rely on other causes of death. We also plan to evaluate agreement for additional causes of death, including stroke, a condition suspected to be underreported in New York City; the 2010 New York City age-adjusted rate of stroke as cause of death was 18.8 per 100,000 compared with a national rate of 39.1 per 100,000 (24,30). Given the potential for these linked data to inform clinical and data-quality improvement, more work should be done to validate hospital discharge records against the medical record, an area of current research at DOHMH.

The linked death certificate–hospital discharge data set permitted evaluation of the effect of the 2009 New York City DOHMH cause-of-death training intervention without a resource-intensive medical record audit. Other vital records jurisdictions with access to linked data should consider using agreement measures to evaluate the quality of cause-of-death data and take action as needed (3). Jurisdictions without linked data should continue to address barriers preventing them from obtaining these linkages. Researchers depending on mortality data should work with vital records jurisdictions to understand data-quality concerns and support quality-improvement interventions for cause-of-death reporting to enhance their own work.
